# A Smart Floating Gate Transistor with Two Control Gates for Active Noise Control

**DOI:** 10.3390/mi10110722

**Published:** 2019-10-25

**Authors:** Cheng Mao, Cheng Yang, Haowen Ma, Feng Yan, Limin Zhang

**Affiliations:** School of Electronic Science and Engineering, Nanjing University, Nanjing 210023, China; 13771683113@163.com (C.M.); njuyangcheng@163.com (C.Y.); mahaowen2012@163.com (H.M.); fyan@nju.edu.cn (F.Y.)

**Keywords:** floating gate transistor, control gate, CMOS device, active noise control

## Abstract

A smart floating gate transistor with two control gates was proposed for active noise control in bioelectrical signal measurement. The device, which is low cost and capable of large-scale integration, was implemented in a standard single-poly complementary metal–oxide–semiconductor (CMOS) process. A model of the device was developed to demonstrate the working principle. Theoretical analysis and simulation results proved the superposition of the two control gates. A series of test experiments were carried out and the results showed that the device was in accordance with the basic electrical characteristics of a floating gate transistor, including the current–voltage (I–V) characteristics and the threshold characteristics observed on the two control gates. Based on the source follower circuit, the experimental results proved that the device can reduce interference by more than 29 dB, which demonstrates the feasibility of the proposed device for active noise control.

## 1. Introduction

**Figure 1:**
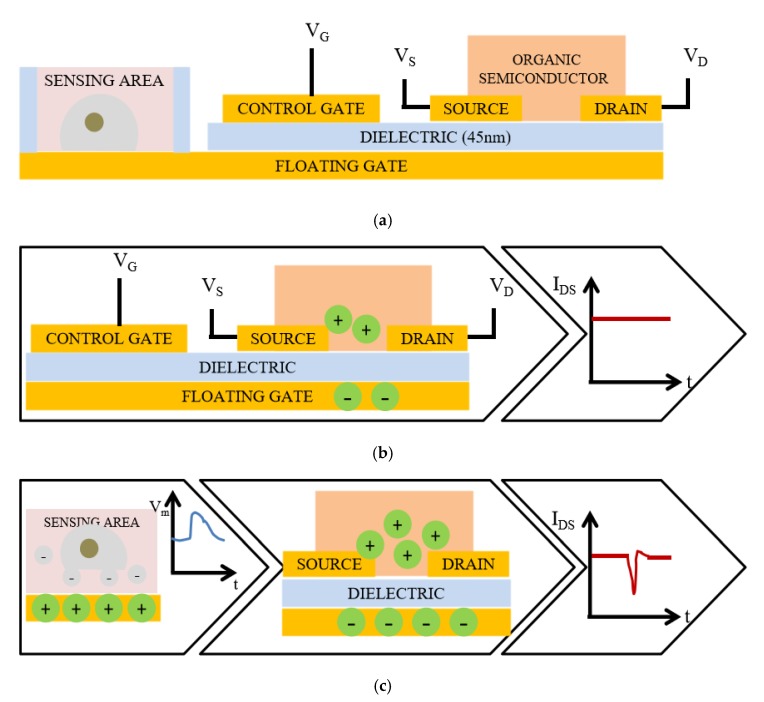
Working principle of a floating gate organic charge-modulated field-effect transistor (OCMFET). (**a**) Cross section of the device, where the floating gate is exposed to the surrounding bioelectrochemical environment as the sensing area. (**b**) The setting of the working point by applying the appropriate $V_{G}$. (**c**) The charge sensing and modulation principle of the device, where the charge variations occurring in close proximity to the sensing area cause a charge separation in the floating gate, which leads to modulation of the charge carrier density inside the channel of the transistor and variation of the output current.

There is growing interest in detecting chemical or bioelectrical signals with solid-state sensors in a complementary metal–oxide–semiconductor (CMOS) process [1,2]. One of the major classes of solid-state sensors is based on the field-effect transistor (FET) [3,4,5]. The multiparametric nature and intrinsic signal amplification ability of FETs make them capable of achieving well beyond what is possible with two terminal devices. The ion-sensitive field-effect transistor (ISFET) [6,7] is an important branch of FET and has attracted great research interest regarding chemical signal detection due to its small size, capability of mass fabrication, and fast response time. The organic thin-film transistor (OTFT) [8,9,10] is another meaningful branch and has been extensively studied for bioelectrical signal detection. For example, a sensing platform based on floating gate OTFTs is used for bioelectrical signal sensing [11,12,13,14]. The working principle of a floating gate organic charge-modulated field-effect transistor (OCMFET) is shown in Figure 1a, where the sensing area is part of the floating gate exposed to the surrounding bioelectrochemical environment to detect signals, and the control gate is used to set the working point with the control capacitor, as shown in Figure 1b. Ionic or cell charge variations occurring in close proximity to the sensing area cause a charge separation in the floating gate, which leads to a modulation of the charge carrier density inside the channel of the transistor, as shown in Figure 1c [15]. The OCMFET device can detect signals in the frequency range of cell electrical activity (10–1000 Hz) and can work without any external reference electrode. However, the floating gate and sensing area are integrated as a whole and can only detect charge signals, which is not suitable for detecting voltage signals because the voltage signal on the floating gate fixes the working point of the device. In addition, it is difficult for the device to suppress interference, which is important for small signal detection.

In order to detect voltage signals, some modifications must be made, such as separating the sensing area and the floating gate. Moreover, the detected signal is accompanied by some interference and the interference can also modulate the channel charge. To suppress the interference actively, the active noise control (ANC) concept has been introduced, which cancels the unwanted primary interference by using a secondary source for signal superposition [16,17,18]. Generally, two input ports are required to achieve active noise control, where one input port is used for primary source detection and another for secondary source input [19]. Therefore, we have proposed a smart floating gate transistor with two control gates (called an ANC device), which is characterized by active noise control and a low-cost standard CMOS process.

Firstly, the physical model of the proposed device was developed to demonstrate the working principle, and the layout of the device was implemented in a standard single-poly CMOS process. Secondly, simulation results are illustrated to show the ANC performance based on the proposed device. Finally, the experimental electrical characterizations of the fabricated device are provided, which demonstrate the feasibility of the device for active noise control applications.

## 2. ANC Device

### 2.1. Device Structure

**Figure 2:**
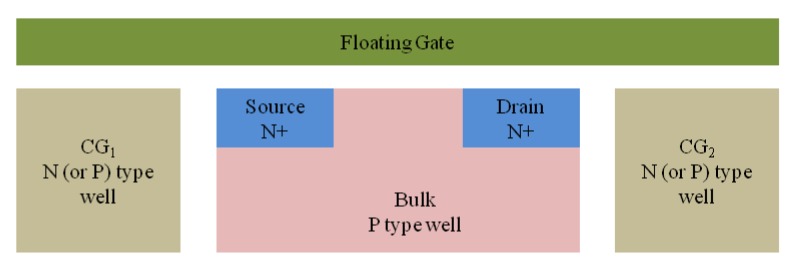
Cross section of the proposed active noise control (ANC) device based on an evolution of a single-poly metal–oxide–semiconductor field-effect transistor (MOSFET), including a source region (S), a drain region (D), a bulk region (B), a floating gate (FG), and two control gates (${\mathrm{CG}}_{1}$ and ${\mathrm{CG}}_{2}$).

The proposed ANC device is based on an evolution of a single-poly metal–oxide–semiconductor field-effect transistor (MOSFET) and the cross section of the proposed ANC device is shown in Figure 2, including a source region (S), a drain region (D), a bulk region (B), a floating gate (FG), and two control gates (${\mathrm{CG}}_{1}$ and ${\mathrm{CG}}_{2}$). Two heavily doped N+ regions are generated on a P-type bulk to serve as the source and the drain, and two N- or P-type physically isolated wells are generated beside the bulk to serve as the two control gates ${\mathrm{CG}}_{1}$ and ${\mathrm{CG}}_{2}$, respectively. The floating gate is generated by a single-poly layer, which is isolated from the bulk and the two control gates by a dielectric layer. The floating gate overlaps the bulk and the two control gates, which leads to the coupling capacitors. The channel current of the proposed device is controlled with two control gates by the coupling capacitors with the floating gate. In application, control gates ${\mathrm{CG}}_{1}$ and ${\mathrm{CG}}_{2}$ are connected to two standard PADs to receive voltage signals. The source, drain, and bulk are connected to three standard PADs for the normal working condition setting.

### 2.2. Device Model 

**Figure 3:**
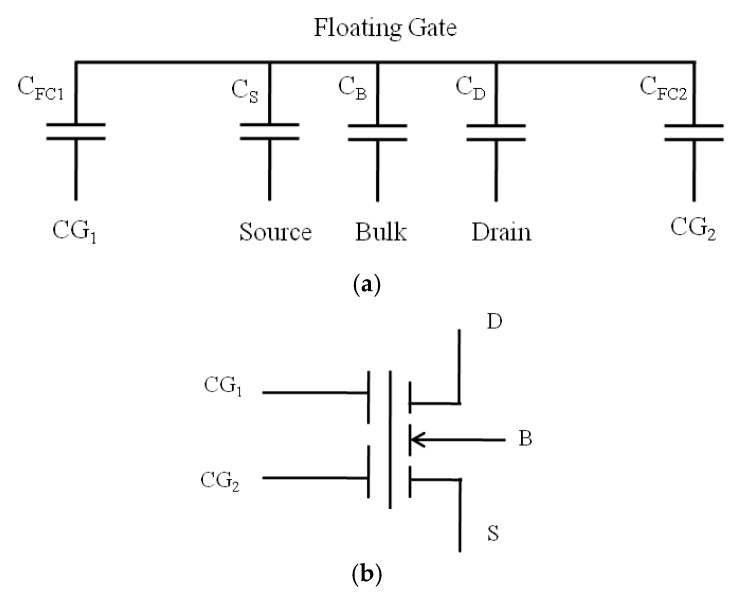
(**a**) The simple model of the equivalent schematic diagram of the proposed ANC device, where $C_{{\mathrm{FC}}_{1}},C_{{\mathrm{FC}}_{2}},C_{S},C_{D},{\;\mathrm{and}\;C}_{B}$ are the capacitors between the floating gate and the control gate ${\mathrm{CG}}_{1}$, the control gate ${\mathrm{CG}}_{2}$, the source, the drain, and the bulk region, respectively. (**b**) Symbols for the ANC device with five terminals.

The equivalent schematic diagram of the proposed ANC device is shown in Figure 3a, where $C_{{\mathrm{FC}}_{1}},C_{{\mathrm{FC}}_{2}},C_{S},C_{D},{\;\mathrm{and}\;C}_{B}\;$ are the capacitors between the floating gate and the control gate ${\mathrm{CG}}_{1}$, the control gate ${\mathrm{CG}}_{2}$, the source, the drain, and the bulk region, respectively. Considering that the charge Q of the floating gate should be equal to 0, the simple model of the proposed device is expressed in Equation (1) [20]:

(1) $$Q=0=C_{FC1}(V_{FG}-V_{CG1})+C_{FC2}(V_{FG}-V_{CG2})+C_{S}(V_{FG}-V_{S})+C_{D}(V_{FG}-V_{D})+C_{B}(V_{FG}-V_{B})$$

where $V_{\mathrm{FG}}$ is the potential on the floating gate; $V_{{\mathrm{CG}}_{1}}$ is the potential on the control gate ${\mathrm{CG}}_{1}$; $V_{{\mathrm{CG}}_{2}}$ is the potential on the control gate ${\mathrm{CG}}_{2}$; and $V_{S}$, $V_{D}$, and $V_{B}\;$ are the potentials on the source, the drain, and the bulk, respectively. Defining the total capacitors $C_{T}$ as the sum of the capacitors of $C_{{\mathrm{FC}}_{1}}$, $C_{{\mathrm{FC}}_{2}}$,$C_{S}$, $C_{D}$, and $C_{B}$, the potential on the floating gate due to capacitive coupling can be expressed as

(2) $$V_{FG}=\frac{C_{FC1}}{C_{T}}V_{CG1}+\frac{C_{FC2}}{C_{T}}V_{CG2}+\frac{C_{S}}{C_{T}}V_{S}+\frac{C_{D}}{C_{T}}V_{D}+\frac{C_{B}}{C_{T}}V_{B}.$$

For $C_{S}$ and $C_{D}$ being far less than $C_{T}$, and the bulk being grounded, Equation (2) can be simplified as

(3) $$V_{FG}=\frac{C_{FC1}}{C_{T}}V_{CG1}+\frac{C_{FC2}}{C_{T}}V_{CG2}.$$

Generally, ${\mathrm{CG}}_{1}$ and ${\mathrm{CG}}_{2}$ are equivalent in terms of electrical characteristics and one of them is always set to zero for measurement of the threshold voltage, so the threshold voltages and conductivity factors of the floating gate and control gates ${\mathrm{CG}}_{1}$ and ${\mathrm{CG}}_{2}$ satisfy the following relationships:

(4) $$V_{T}^{FG}=\frac{C_{FC1}}{C_{T}}V_{T}^{CG1}=\frac{C_{FC2}}{C_{T}}V_{T}^{CG2}$$

(5) $$\beta^{FG}=\frac{C_{T}}{C_{FC1}}\beta^{CG1}=\frac{C_{T}}{C_{FC2}}\beta^{CG2}$$

where $V_{T}^{\mathrm{FG}}$ is the threshold for the floating gate, $V_{T}^{{\mathrm{CG}}_{1}}$ is the threshold for control gate ${\mathrm{CG}}_{1}$, $V_{T}^{{\mathrm{CG}}_{2}}$ is the threshold for control gate ${\mathrm{CG}}_{2}$, $\beta^{\mathrm{FG}}$ is the conductivity factor for the floating gate, $\beta^{{\mathrm{CG}}_{1}}$ is the conductivity factor for control gate ${\mathrm{CG}}_{1}$, and $\beta^{{\mathrm{CG}}_{2}}$ is the conductivity factor for control gate ${\mathrm{CG}}_{2}$.

Accordingly, the transformed current–voltage (I–V) equations of the proposed ANC device in the triode region (TR) and the saturation region (SR) can be expressed by

(6) $$\mathrm{TR}\;\left|V_{DS}\right|<\frac{C_{FC1}}{C_{T}}\left|V_{CG1}+\frac{C_{\mathrm{FC}2}}{C_{FC1}}V_{CG2}-\frac{C_{T}}{C_{FC1}}V_{S}-V_{T}^{CG1}\right|\;I_{D}=\beta^{CG1}\left[\left(V_{CG1}+\frac{C_{FC2}}{C_{FC1}}V_{CG2}-\frac{C_{T}}{C_{FC1}}V_{S}-V_{T}^{CG}\right)V_{DS}-\frac{1}{2}\frac{C_{T}}{C_{FC1}}V_{DS}^{2}\right]$$

(7) $$\mathrm{SR}\;\left|V_{DS}\right|\geq \frac{C_{FC1}}{C_{T}}\left|V_{CG1}+\frac{C_{\mathrm{FC}2}}{C_{FC1}}V_{CG2}-\frac{C_{T}}{C_{FC1}}V_{S}-V_{T}^{CG1}\right|\;I_{D}=\frac{\beta^{CG1}}{2}\left(\frac{C_{FC1}}{C_{T}}\right){\left(V_{CG1}+\frac{C_{FC2}}{C_{FC1}}V_{CG2}-\frac{C_{T}}{C_{FC1}}V_{S}-V_{T}^{CG1}\right)}^{2}$$

which show the relationship between $I_{D}$ and $V_{{\mathrm{CG}}_{1}}$, $V_{{\mathrm{CG}}_{2}}$, $V_{S}$, and $V_{D}$. In Equation (7), $I_{D}$ in the saturation region is not affected by $V_{\mathrm{DS}}$, which is greatly convenient for reading the output signal of the ANC device. Assuming $I_{D}$ to be a constant value and the source voltage $V_{S}$ to be the output signal, the signal of the control gate is easily obtained by measuring the output voltage $V_{S}$. This readout method is called the source follower method, where the voltage of source $V_{S}$ can be expressed by the voltage of two control gates as

(8) $$V_{S}=\frac{C_{FC1}}{C_{T}}V_{CG1}+\frac{C_{FC2}}{C_{T}}V_{CG2}-\sqrt{\frac{2I_{D}}{\beta^{CG1}}\left(\frac{C_{FC1}}{C_{T}}\right)}-\frac{C_{FC1}}{C_{T}}V_{T}^{CG1}.$$

Normally, one control gate is used to set the DC operation point of the device and works as the secondary source input, and the other control gate is used to detect the effective signal with background interference. The subsequent circuits extract the interference from the output signal $V_{S}$ and actively generate an inverse interference for secondary input. Thus, the secondary interference on one control gate and the primary interference on the other control gate work together to cancel each other and, finally, output the effective signal in $V_{S}$, with the interference being as small as possible. 

According to the equivalent schematic diagram of the proposed ANC device shown in Figure 3a and the standard symbols for MOS transistors, the symbols for the proposed ANC device are shown in Figure 3b, which has five terminals of drain (D), source (S), bulk (B), control gate (${\mathrm{CG}}_{1}$), and control gate (${\mathrm{CG}}_{2}$).

### 2.3. Device Layout

**Figure 4:**
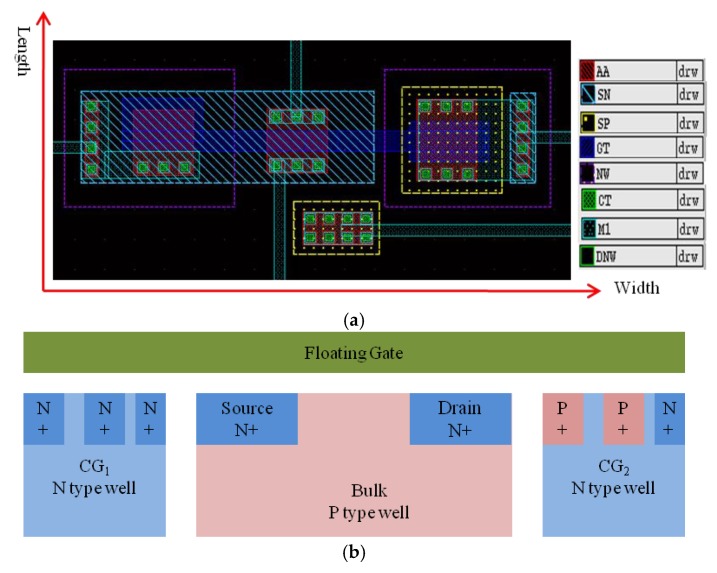
(**a**) Layout of the proposed ANC device, where AA is the active area, SN is the N+ implantation for the source and drain, SP is the P+ implantation, GT is the polysilicon gate, NW is the N-type well, DNW is the deep N-type well, CT is the contact area, and M1 is the metal one. (**b**) Cross section of the layout, where ${\mathrm{CG}}_{1}$ is a N-MOSC and ${\mathrm{CG}}_{2}$ is a P-MOSFET with a common source, drain, and substrate.

The proposed ANC device was implemented in a standard 0.18 μm single-poly CMOS process. Figure 4 shows the layout and cross section of the proposed ANC device, where AA is the active area, SN is the N+ implantation for source and drain, SP is the P+ implantation, GT is the polysilicon gate, NW is the N-type well, DNW is the deep N-type well, CT is the contact area, and M1 is the metal one. As shown in Figure 4, two control gates were formed in two N-type wells without special isolation from the P-type bulk. ${\mathrm{CG}}_{1}$ is an N-MOSC, while ${\mathrm{CG}}_{2}$ is a P-MOSFET with a common source, drain, and substrate. The two different structures both modulated the charges in the floating gate by the coupling capacitors. The coupling capacitor was determined by the overlap area between the floating gate and the control gate. The design parameters of the ANC device are listed in Table 1, where L is the abbreviation for the length and W is the abbreviation for the width of the areas shown in Figure 4a.

## 3. Simulation Results

**Figure 5:**
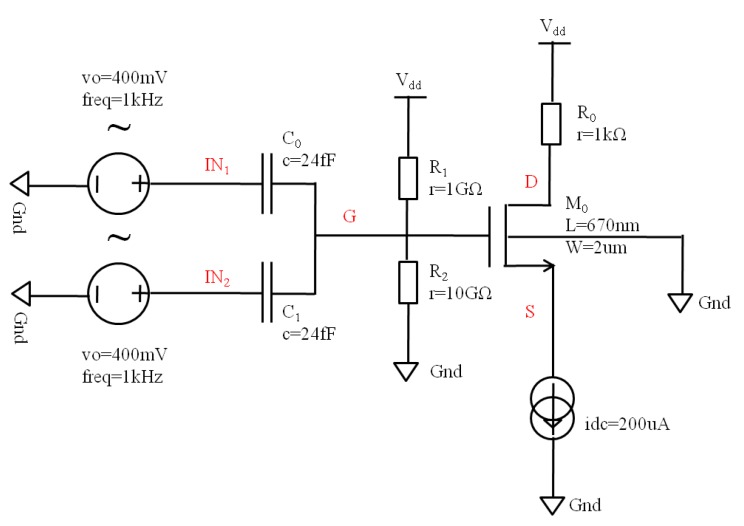
A simulation schematic based on the proposed ANC device with a standard MOSFET $M_{0}$ and two capacitors $C_{0}$ and $C_{1}$, where capacitors $C_{0}$ and $C_{1}$ can be seen as the coupling capacitors $C_{{\mathrm{FC}}_{1}}$ and $C_{{\mathrm{FC}}_{2}}$; MOSFET $M_{0}$ represents the combination of the floating gate, the source, the drain, and the p-bulk; inputs ${\mathrm{IN}}_{1}$ and ${\mathrm{IN}}_{2}$ are equivalent to the control gates ${\mathrm{CG}}_{1}$ and ${\mathrm{CG}}_{2}$; and two resistors $R_{1}$ and $R_{2}$ are used to provide the bias potential for the gate.

**Figure 6:**
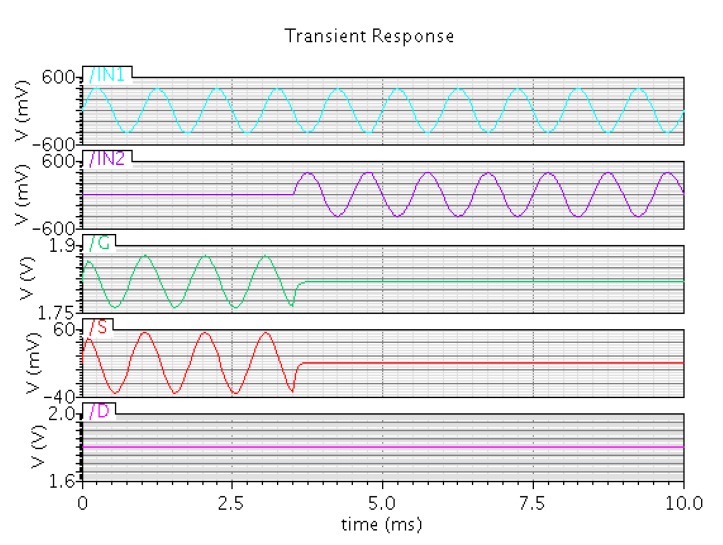
Simulation result of the proposed ANC device, where signals ${\mathrm{IN}}_{1}$, ${\mathrm{IN}}_{2}$, G, S, and D are the measured voltages from the red node ${\mathrm{IN}}_{1},$ ${\mathrm{IN}}_{2}$, G, S, and D shown in Figure 5, and signals ${\mathrm{IN}}_{1}$ and ${\mathrm{IN}}_{2}$ are simulated as the primary interference and secondary interference, respectively. From 0 to 3.5 ms, only signal ${\mathrm{IN}}_{1}$ modulates the gate voltage with a certain attenuation and the source voltage follows the gate voltage. After 3.5 ms, signals ${\mathrm{IN}}_{1}$ and ${\mathrm{IN}}_{2}$ both modulate the gate voltage and the source voltage becomes a DC voltage without noise.

A simulation schematic based on the proposed ANC device is shown in Figure 5. The device is equivalent to a standard MOSFET $M_{0}$ and two capacitors $C_{0}$ and $C_{1}$, where capacitors $C_{0}$ and $C_{1}$ can be seen as the coupling capacitors $C_{{\mathrm{FC}}_{1}}$ and $C_{{\mathrm{FC}}_{2}}$, and MOSFET $M_{0}$ is equivalent to the combination of the floating gate, the source, the drain, and the p-bulk. Inputs ${\mathrm{IN}}_{1}$ and ${\mathrm{IN}}_{2}$ are equivalent to the control gates ${\mathrm{CG}}_{1}$ and ${\mathrm{CG}}_{2}$. Two resistors $R_{1}$ and $R_{2}$ were used to provide the bias potential for the gate. The device was operated in the source follower mode under the saturation state. The values of two capacitors $C_{0}$ and $C_{1}$ were set to 24 fF according to the parameters of ${\mathrm{CG}}_{1}$ and ${\mathrm{CG}}_{2}$, as shown in Table 1, calculated as Equation (9). The saturation current based on the standard MOSFET $M_{0}$ was calculated as Equation (10), where $V_{\mathrm{GS}}$ is the potential difference between the gate and the source, and $V_{T}$ is the threshold for the MOSFET device. Signals ${\mathrm{IN}}_{1}$ and ${\mathrm{IN}}_{2}$ are two sinusoidal wave signals with inverse phase and the same frequency and amplitude. The voltages of nodes G, D, and S were measured and the results are shown in Figure 6. From 0 to 3.5 ms, signal ${\mathrm{IN}}_{2}$ was a DC signal, only signal ${\mathrm{IN}}_{1}$ modulated the gate voltage with a certain attenuation, and the source voltage followed the gate voltage. After 3.5 ms, signals ${\mathrm{IN}}_{1}$ and ${\mathrm{IN}}_{2}$ both modulated the gate voltage. When the primary interference was detected by input end ${\mathrm{IN}}_{1}$, the source follower structure output the similar primary interference, and subsequent circuits actively generated an inverse secondary interference for input end ${\mathrm{IN}}_{2}$, which led to an effective signal without the primary interference at the source output by the superposition of two input signals. As a result, the source voltage was a DC voltage without the input sinusoidal signal, which showed the good performance of active noise control.

(9) $$C=WLC_{ox}^{’}=WL\frac{\varepsilon_{r(\mathrm{ox})}\varepsilon_{0}}{t_{ox}}=1.35\times 10^{-4}cm\times 2\times 10^{-4}cm\times \frac{3.9\times (8.85\times 10^{-14}F/cm)}{4\times 10^{-7}cm}=23.3fF$$

(10) $$\begin{array}{l} I_{D}=\frac{W\mu C_{ox}^{’}}{2L}{\left(V_{GS}-V_{T}\right)}^{2}\\ =\frac{2\times 10^{-4}cm\times 500{\mathrm{cm}}^{2}/V\cdot s}{2\times 0.67\times 10^{-4}cm}\times \frac{3.9\times (8.85\times 10^{-14}F/cm)}{4\times 10^{-7}cm}\times {\left(1.8V-0.5V-0.7V\right)}^{2}\\ =232\mu A \end{array}$$

## 4. Experimental Results

### 4.1. I–V Characteristics

**Figure 7:**
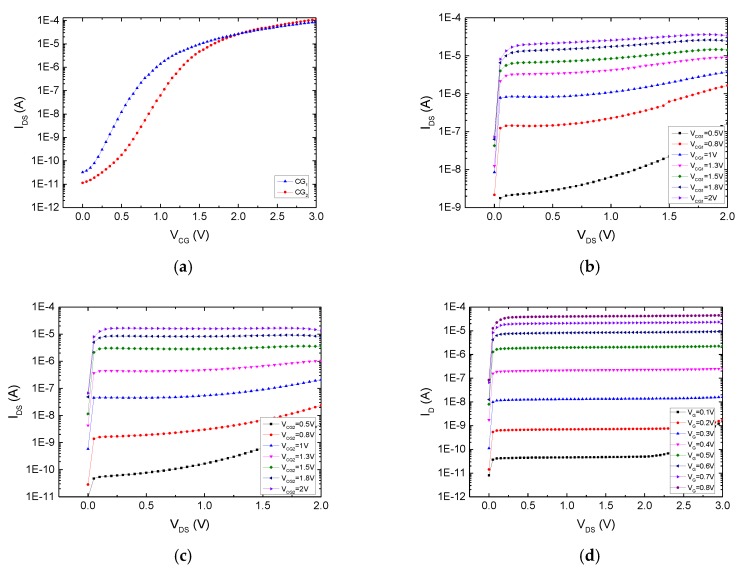
Current–voltage (I–V) characteristics of an ANC device and a standard MOSFET. (**a**) The I_D_–V_G_ characteristics of an ANC device show that each control gate can modulate the device and the threshold voltages are within a reasonable range. (**b**) I_D_–V_D_ characteristics for control gate ${\mathrm{CG}}_{1}$. (**c**) I_D_–V_D_ characteristics for control gate ${\mathrm{CG}}_{2}$. (**d**) I_D_–V_D_ characteristics of a standard MOSFET.

The proposed ANC device was fabricated in a 0.18 μm single-poly CMOS process and the performance was measured with the Keithley 4200 Semiconductor Characterization System. To test the capability of each control gate to modulate the charge carrier density of the device channel, one of the two control gates was set to zero voltage and the other was input with a scanning voltage, while the source and the bulk were grounded and the drain was set to 0.5 V. Figure 7a shows the I_D_–V_G_ characteristics of the device, where the blue line is for control gate ${\mathrm{CG}}_{1}$ and the red line is for control gate ${\mathrm{CG}}_{2}$, which shows that each control gate was able to modulate the device and the threshold voltages were within a reasonable range.

Figure 7b and c show the I_D_–V_D_ curves for control gates ${\mathrm{CG}}_{1}$ and ${\mathrm{CG}}_{2}$, respectively. When the control gate voltage was not large enough, a high drain voltage caused a secondary increase of the drain current. For comparison, the I_D_–V_D_ curve for a standard MOSFET with the same gate size as that of the ANC device is shown in Figure 7d. It can be seen that when the gate voltage of the standard MOSFET is less than 0.2 V, the current $I_{D}$ increases again after saturation, which is a characteristic similar to that of the proposed device. This may be caused by reverse breakdown of the pn junction between the drain and the substrate because most of the increasing current comes from the substrate end, which can easily happen when the gate voltage is far below the threshold voltage. When the gate voltage becomes higher, a deeper depletion region is generated below the channel, which connects to the depletion region below the drain region and protects the pn junction from breakdown. Fortunately, in active noise control detection applications, the device is always turned on in the saturation state, which will not cause serious secondary increase.

### 4.2. Device Threshold Characteristic 

**Figure 8:**
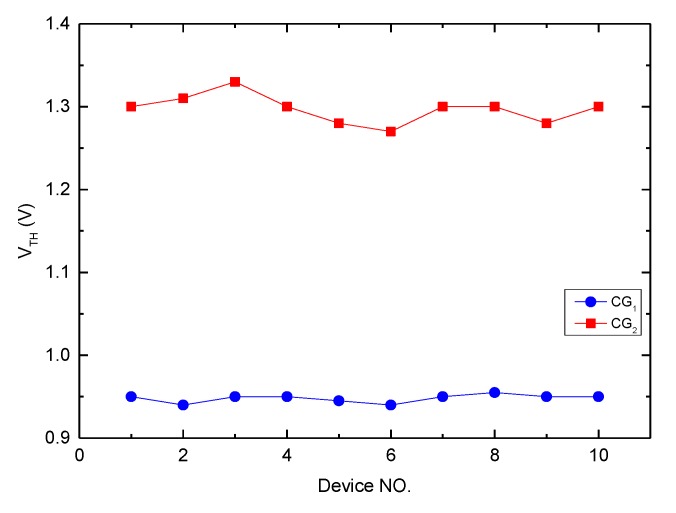
The threshold voltages for two control gates of 10 devices, where the average threshold voltage for ${\mathrm{CG}}_{1}$ is 0.95 V and that for ${\mathrm{CG}}_{1}$ is 1.3 V.

The basic structure of the proposed ANC device was a 1.8 V MOSFET with a channel length of 0.67 μm and a channel width of 2.03 μm. The designed overlap area between control gate ${\mathrm{CG}}_{1}$ and the floating gate was slightly bigger than that between control gate ${\mathrm{CG}}_{2}$ and the floating gate. In the measurement, one of the control gates was grounded and the other was for threshold scanning. The threshold voltage for each control gate is illustrated clearly in Figure 8. The average threshold voltage for ${\mathrm{CG}}_{1}$ was 0.95 V and that for ${\mathrm{CG}}_{2}$ was 1.3 V.

As discussed in the section on the device model, the threshold relationships satisfy Equation (4). According to the parameters in Table 1, the estimated threshold voltages of the two control gates should almost be the same value, but the measurement result was inconsistent. However, it can be seen from the layout that the real capacitor of ${\mathrm{CG}}_{1}$ was larger than that of ${\mathrm{CG}}_{2}$, which explains why the threshold voltage of control gate ${\mathrm{CG}}_{1}$ was smaller than that of control gate ${\mathrm{CG}}_{2}$.

### 4.3. ANC Experimental Verification

**Figure 9:**
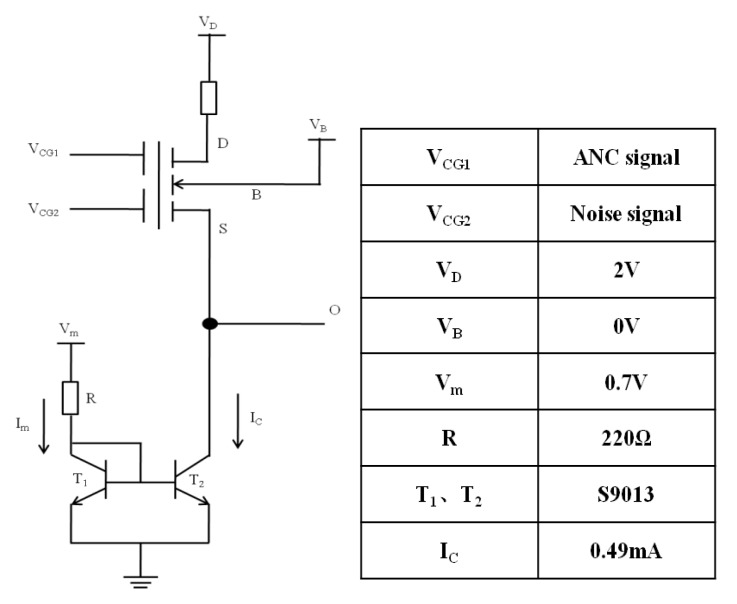
The circuit with source follower mode, where a current mirror, composed of two transistors $T_{1}$ and $T_{2}$ and a resistor R, is used to provide a constant saturation current $I_{C}$, and $V_{D}$ is set to 2 V to ensure the device is in the saturation state.

The circuit with source follower mode is shown in Figure 9, where a current mirror, composed of two transistors $T_{1}$ and $T_{2}$ and a resistor R, was used to provide a constant saturation current $I_{C}$, and $V_{D}$ was set to 2 V to ensure that the device was in the saturation state.

**Figure 10:**
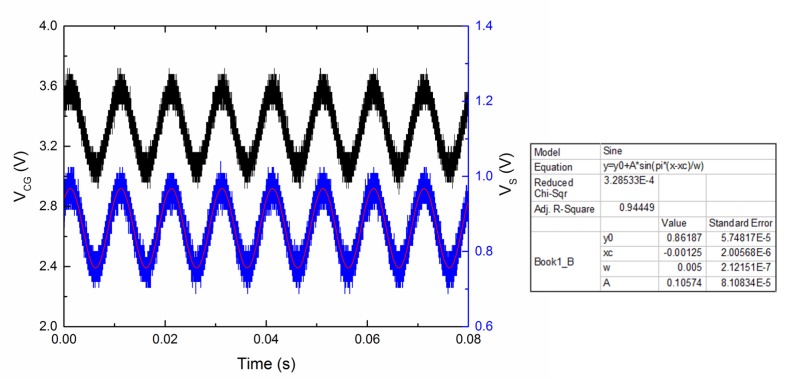
Characterization of device background noise in the circuit, where control gate $CG_{1}$ is applied with a standard sinusoidal signal (with a 3.3 V DC bias) as shown with the black line, and the output signal $V_{S}$ is shown with the blue line and fitted by a sinusoidal function as the red line with the fitting parameters given in the insets. The absolute value of the background noise is less than 0.33 mV.

To characterize the background noise of the proposed ANC device in the circuit system, control gate $CG_{2}$ was grounded and control gate $CG_{1}$ was applied with a standard sinusoidal signal (with a 3.3 V DC bias), shown as the black line in Figure 10. The output signal $V_{S}$ was sampled and analyzed as the blue line, shown in Figure 10. The output signal curve was fitted by a sinusoidal function. The mean square of the residual expressed in Equation (11) represents the background noise of the device in the circuit system, where $V_{\mathrm{bn}}$ is the background noise, N is the number of sampled points, $y_{\mathrm{real}}$ is the sampled output signal, and $y_{\mathrm{fit}}$ is the fitted signal as mentioned above. The absolute value of the background noise was less than 0.33 mV for N being 1000, which shows the feasibility of the device in detecting small biosignals. It should be noted that the background noise can be reduced further by integrating all circuits in the same CMOS process.

(11) $$V_{\mathrm{bn}}=\sqrt{\frac{{\sum_{i=1}^{N}\left(y_{\mathrm{real},i}{\;-\;y}_{\mathrm{fit},i}\right)}^{2}}{N}}.$$

**Figure 11:**
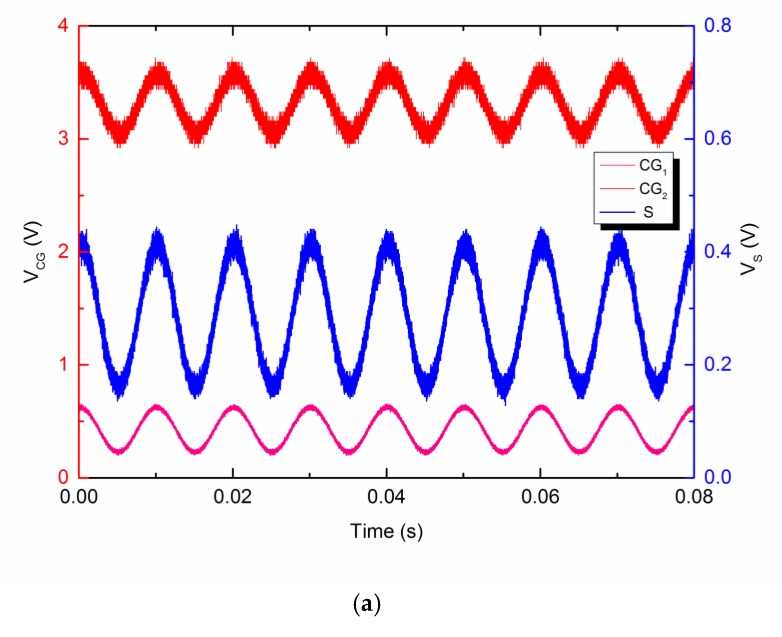

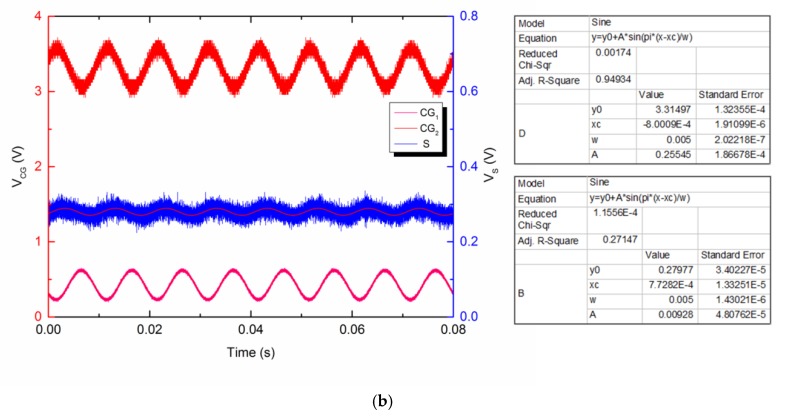
The measured voltage as a function of time, where the pink line is the signal on control gate $CG_{1}$, the red line is the signal on control gate $CG_{2}$, the blue line is the output signal $V_{S}$, and the input interference on control gate $CG_{2}$ and the output signal are fitted by a sinusoidal function respectively with the parameters given in the insets. (**a**) Without active noise control, the output signal $V_{S}$ follows the superposition of signals $V_{CG_{2}}$ and $V_{CG_{1}}$ as calculated. (**b**) With active noise control, the output signal $V_{S}$ is 9 mV and has a 29 dB attenuation for the input interference of 255 mV.

In Figure 11, the pink line is the signal on control gate $CG_{1}$, the red line is the signal on control gate $CG_{2}$ (both corresponding to the left axis), and the blue line is the output signal $V_{S}$, corresponding to the right axis. $V_{CG_{2}}$ is an input sinusoidal wave signal, considered as the primary interference. $V_{CG_{1}}$ is the secondary interference relevant to the primary interference from $V_{CG_{2}}$. The measured signal $V_{S}$ as function of time is shown in Figure 11, which was in good agreement with expectations. As shown in Figure 11a, the output signal $V_{S}$ followed the superposition of signals $V_{CG_{2}}$ and $V_{CG_{1}}$, as calculated in Equation (8). When the active noise control system was turned on, the ANC signal on $CG_{1}$ was adjusted by the subsequent feedback circuits to a signal with an inverse phase from the primary interference on $CG_{2}$. The amplitude of the ANC signal was modified by adjusting the gain of the subsequent circuits manually until the output signal $V_{S}$ became as small as possible. Feedback system design is a large subject area and there are many kinds of specific circuit forms for feedback system implementation. One kind of feedback circuit for an ANC system consists of a bandpass filter and an inverting amplifier. The bandpass filter extracts the interference from the output signal and the inverting amplifier generates an inverse interference for secondary input. Figure 11b shows the result of the ANC system, where the input interference amplitude was 0.255 V and the output interference amplitude after ANC was 0.009 V. Therefore, the circuits can attenuate the interference by greater than 29 dB. The device has proved to be feasible and reliable for active noise control application. Moreover, the device works at ultra-low voltages and without any external reference electrode, and it also provides the capability of large-scale integration at a low cost for fabrication in a standard single-poly CMOS process.

## 5. Conclusions

A smart floating gate transistor with two control gates was proposed for active noise control in bioelectrical signal measurement. A model of the device was developed and analyzed to demonstrate the working principle of the electrical behavior. Theoretical analysis and simulation results proved that the superposition of the two control gates can be reflected at the source end. To verify the feasibility of the proposed ANC device, a device with a novel structure was designed and fabricated in a standard 0.18 μm single-poly CMOS process. A series of test experiments were carried out and the results showed that the devices were in accordance with the basic electrical characteristics of floating gate transistors, including the I–V characteristics and the threshold characteristics observed on two control gates. Based on the source follower circuit, the experimental results proved that the device can reduce interference by more than 29 dB, and that is possesses the outstanding characteristic of low-cost, large-scale integration for fabrication in a standard single-poly CMOS process.

Future work will be directed toward fabricating the readout circuit of the proposed device and the subsequent circuit for secondary input in a standard single-poly CMOS process to enhance the large-scale integration ability and reduce the background noise further.

## Figures and Tables

**Table 1:** The design parameters of the device.

Device	Length (μm)	Width (μm)
CG_1_	1.375	2.01
MOS	0.67	2.03
CG_2_	1.35	2.00

## References

[1] Barbaro M., Bonfiglio A., Raffo L. (2006). A charge-modulated FET for detection of biomolecular processes: conception, modeling, and simulation. IEEE Trans. Electron Devices.

[2] Meyburg S., Stockmann R., Moers J., Offenhäusser A., Ingebrandt S. (2007). Advanced CMOS process for floating gate field-effect transistors in bioelectronic applications. Sens. Actuator B-Chem..

[3] Zhang Q., Subramanian V. (2007). DNA hybridization detection with organic thin film transistors: Toward fast and disposable DNA microarray chips. Biosens. Bioelectron..

[4] Zhang X.H., Lee S.M., Domercq B., Kippelen B. (2008). Transparent organic field-effect transistors with polymeric source and drain electrodes fabricated by inkjet printing. Appl. Phys. Lett..

[5] Kergoat L., Piro B., Berggren M., Pham M.C., Yassar A., Horowitz G. (2012). DNA detection with a water-gated organic field-effect transistor. Org. Electron..

[6] Jimenez-Jorquera C., Orozco J., Baldi A. (2009). ISFET Based Microsensors for Environmental Monitoring. Sensors.

[7] Moser N., Rodriguez-Manzano J., Lande T.S., Georgiou P. (2018). A Scalable ISFET Sensing and Memory Array with Sensor Auto-Calibration for On-Chip Real-Time DNA Detection. IEEE Trans. Biomed. Circuits Syst..

[8] Ogier S.D., Matsui H., Feng L., Simms M., Mashayekhi M., Carrabina J., Terés L., Tokito S. (2018). Uniform, high performance, solution processed organic thin-film transistors integrated in 1 MHz frequency ring oscillators. Org. Electron..

[9] Krammer M., Borchert J.W., Petritz A., Karner-Petritz E., Schider G., Stadlober B., Klauk H., Zojer K. (2019). Critical Evaluation of Organic Thin-Film Transistor Models. Crystals.

[10] Liao C., Yan F. (2013). Organic Semiconductors in Organic Thin-Film Transistor-Based Chemical and Biological Sensors. Polym. Rev..

[11] Lai S., Viola F., Cosseddu P., Bonfiglio A. (2018). Floating Gate, Organic Field-Effect Transistor-Based Sensors towards Biomedical Applications Fabricated with Large-Area Processes over Flexible Substrates. Sensors.

[12] Spanu A., Viola F., Lai S., Cosseddu P., Ricci P.C., Bonfiglio A. (2017). A reference-less pH sensor based on an organic field effect transistor with tunable sensitivity. Org. Electron..

[13] Demelas M., Lai S., Casula G., Scavetta E., Barbaro M., Bonfiglio A. (2012). An organic, charge-modulated field effect transistor for DNA detection. Sens. Actuator B-Chem..

[14] Lai S., Barbaro M., Bonfiglio A. (2016). Tailoring the sensing performances of an OFET-based biosensor. Sens. Actuator B-Chem.

[15] Spanu A., Lai S., Cosseddu P., Tedesco M., Martinoia S., Bonfiglio A. (2015). An organic transistor-based system for reference-less electrophysiological monitoring of excitable cells. Sci. Rep..

[16] Zhang L., Tao J., Qiu X. (2012). Active control of transformer noise with an internally synthesized reference signal. J. Sound Vib..

[17] Zhang L., Wu L., Qiu X. (2013). An intuitive approach for feedback active noise controller design. Appl. Acoust..

[18] Gan W.S., Mitra S., Kuo S.M. (2005). Adaptive feedback active noise control headset: implementation, evaluation and its extensions. IEEE Trans. Consum. Electron..

[19] Tseng W.K., Rafaely B., Elliott S.J. (1998). Combined feedback–feedforward active control of sound in a room. J. Acoust. Soc. AM..

[20] Pavan P., Bez R., Olivo P., Zanoni E. (1997). Flash memory cells-an overview. Proc. IEEE.

